# 1k-RiCA (*1K-Rice Custom Amplicon*) a novel genotyping amplicon-based SNP assay for genetics and breeding applications in rice

**DOI:** 10.1186/s12284-019-0311-0

**Published:** 2019-07-26

**Authors:** Juan David Arbelaez, Maria Stefanie Dwiyanti, Erwin Tandayu, Krizzel Llantada, Annalhea Jarana, John Carlos Ignacio, John Damien Platten, Joshua Cobb, Jessica Elaine Rutkoski, Michael J. Thomson, Tobias Kretzschmar

**Affiliations:** 1 International Rice Research Institute, DAPO Box 7777, 1301 Los Baños, Metro Manila Philippines; 2 Research Faculty of Agriculture, Hokkaido University, Sapporo, Hokkaido 060-8589 Japan; 3 Department of Soil and Crop Sciences, Texas A&M University, College Station, Houston, TX 77843 USA; 4 Southern Cross Plant Sciences, Southern Cross University, PO Box 157, Lismore, NSW 2480 Australia

**Keywords:** Single nucleotide polymorphism (SNP), *Oryza sativa*, *Indica*, SNP fingerprinting, Genomic selection, Marker-assisted selection (MAS), Amplicon-based next generation sequencing, Breeding and genotyping

## Abstract

**Background:**

While a multitude of genotyping platforms have been developed for rice, the majority of them have not been optimized for breeding where cost, turnaround time, throughput and ease of use, relative to density and informativeness are critical parameters of their utility. With that in mind we report the development of the *1K-Rice Custom Amplicon*, or 1k-RiCA, a robust custom sequencing-based amplicon panel of ~ 1000-SNPs that are uniformly distributed across the rice genome, designed to be highly informative within *indica* rice breeding pools, and tailored for genomic prediction in elite *indica* rice breeding programs.

**Results:**

Empirical validation tests performed on the 1k-RiCA showed average marker call rates of 95% with marker repeatability and concordance rates of 99%. These technical properties were not affected when two common DNA extraction protocols were used. The average distance between SNPs in the 1k-RiCA was 1.5 cM, similar to the theoretical distance which would be expected between 1,000 uniformly distributed markers across the rice genome. The average minor allele frequencies on a panel of *indica* lines was 0.36 and polymorphic SNPs estimated on pairwise comparisons between *indica* by *indica* accessions and *indica* by *japonica* accessions were on average 430 and 450 respectively. The specific design parameters of the 1k-RiCA allow for a detailed view of genetic relationships and unambiguous molecular IDs within *indica* accessions and good cost vs. marker-density balance for genomic prediction applications in elite *indica* germplasm. Predictive abilities of Genomic Selection models for flowering time, grain yield, and plant height were on average 0.71, 0.36, and 0.65 respectively based on cross-validation analysis. Furthermore the inclusion of important trait markers associated with 11 different genes and QTL adds value to parental selection in crossing schemes and marker-assisted selection in forward breeding applications.

**Conclusions:**

This study validated the marker quality and robustness of the 1k-RiCA genotypic platform for genotyping populations derived from *indica* rice subpopulation for genetic and breeding purposes including MAS and genomic selection. The 1k-RiCA has proven to be an alternative cost-effective genotyping system for breeding applications.

**Electronic supplementary material:**

The online version of this article (10.1186/s12284-019-0311-0) contains supplementary material, which is available to authorized users.

## Background

Rice (*Oryza sativa*) is the staple food for more than 3.5 billion people (Wang et al. 2018). To meet the future global demand for rice an estimated additional 116 million tons of it will be needed by 2035 (Seck et al. 2012). Based on this assumption up to 1.5 to 2.4% yield increase per year will have to be attained despite limiting water supplies, reduced cultivation area, and fluctuating climatic conditions (Seck et al. 2012; Ray et al. 2013). Recent studies indicated that rice yield increases have plateaued in different regions of the world (Ray et al. 2013). Overcoming this yield stagnation requires great efforts and innovation to improve the efficiencies in cultivation, management and, not least of all, varietal development. Rice breeders and geneticists need to capitalize on the latest methods and tools to accelerate variety development and increase the annual rate of genetic gains for multiple traits to maintain a stable food supply that meets the needs of a growing population (Thomson et al. 2017).

Recent advances in next-generation sequencing (NGS) and single nucleotide polymorphism (SNP) genotyping promise to accelerate crop improvement provided they are properly integrated and deployed into breeding programs (Thomson 2014). SNPs are the markers of choice for most high-throughput genotyping applications. They are abundant, co-dominant and evenly distributed along the genome. High-throughput SNP genotyping platforms have enabled rapid, routine and cost effective genotyping solutions for targeted marker assisted selection (MAS) on large effect QTLs or genes of interest (Ramkumar et al. 2015; Kurokawa et al. 2016). Genome-wide SNP genotyping combined with effective and precise sample -tracking, −collection, and -DNA extraction is a powerful tool that can reshape breeding programs and facilitates increasing gain from selection. Genome-wide SNP genotyping enables integrating targeted MAS and genomic selection (GS) approaches into different breeding strategies and contributes to increasing the efficiency of multiple other breeding activities (Chen et al. 2016), such as seed purity testing, pedigree verification, and varietal identification (Tian et al. 2015).

NGS and array-based technologies are the two dominant SNP detection systems for genome-wide genotyping. NGS methods, commonly termed genotyping-by-sequencing (GBS), range from whole genome re-sequencing (or skim sequencing) (Scheben et al. 2018) to reduced representation sequencing (RRS) (Elshire et al. 2011). GBS-type technologies have been applied to a range of crops, providing large data volume at a low cost per data point and are independent of prior genomic information, genome size, genome organization or ploidy (Elshire et al. 2011). In rice, GBS has been used to characterize bi-parental populations (Spindel et al. 2013; Arbelaez et al. 2015), multi-parent mapping populations (Bandillo et al. 2013), nested association mapping populations (Fragoso et al. 2017), and breeding populations (Begum et al. 2015; Spindel et al. 2015). Although NGS platforms have the advantage of minimal ascertainment bias, they currently require complex experimental protocols, sophisticated data analysis, and bioinformatics pipelines to process raw sequence data into useful genotypic matrices. This added capacity cost currently limits the applicability of NGS platforms in many public breeding programs (Chen et al. 2014).

Array-based genotyping platforms provide high- medium- and low-density genome scans, robust high-quality allele calling, easy handling, and simplified analysis to routinely generate genotypic datasets (Rasheed et al. 2017; Scheben et al. 2018). Their main disadvantages are lack of flexibility due to ascertainment bias, and, despite a reasonable low cost per data point, a comparatively high cost per sample (Rasheed et al. 2017). A number of low-, medium-, and high-resolution SNP arrays have been developed for rice and their utility was demonstrated across a range of applications. In the low and medium density range this includes, but is not limited to, the 384-plex BeadXpress (Chen et al. 2011), the GoldenGate 1536 SNPs (Zhao et al. 2010) and two Illumina Infinium-based 6 K arrays, the RiceSNP6K (Yu et al. 2014) and the C6AIR (Thomson et al. 2017). They have been used for diversity analysis, QTL mapping, marker assisted backcrossing (MABC) and pedigree verification among breeding lines. On the high density end, the 700 K High Density Rice Array (HDRA700K) (McCouch et al. 2016), two 50 K arrays (RiceSNP50K and Affymatrix 50 K) (Chen et al. 2014; Singh et al. 2015) and a 44 K array (GeneChip Rice 44 K) (Zhao et al. 2011) were deployed mainly for genome-wide association studies (GWAS) (Famoso et al. 2011; Crowell et al. 2016). These arrays were developed to be highly informative across diverse germplasm including different rice subpopulations and they were optimized to dissect (phylo-) genetic relationships and phenotype to genotype associations.

Less effort has been made in developing informative high-throughput, and cost effective genotyping solution specifically designed for applied breeding programs. Large-scale application in rice breeding with emphasis on population improvement strategies that integrate GS, necessitates routine genotyping of thousands of lines per season at the shortest turnaround time possible to make in-season decisions based on genomic estimated breeding values (GEBV). Globalized breeding programs require breeding populations to be evaluated at multiple locations with different planting dates leaving a very small window to sample, process and analyze genotypic data. The current complexity of GBS technologies and cost of array technologies are limiting in this context.

GS approaches are increasingly being adopted to accelerate the rate of genetic improvement of key agriculturally important species, including rice (Spindel et al. 2015; Grenier et al. 2015; Monteverde et al. 2018). GS uses a ‘training population’ of individuals that have been both genotyped and phenotyped to develop a model that takes genotypic data from a ‘candidate population’ of untested individuals and produces GEBVs (Jannink et al. 2010). A major challenge in implementing GS in most plant breeding programs is the cost of genotyping per sample. The expected value of the information gained by genotyping must exceed the cost of obtaining the genotypes (Boichard et al. 2012). Studies that have evaluated the effect of the number of markers on GS accuracies in closely related breeding germplasm consistently show diminishing returns from increasing marker number (Rutkoski et al. 2013; Gorjanc et al. 2017; Raoul et al. 2017). Provided high levels of informativeness at each target locus, genotyping at relatively low density could be an effective way to reduce cost with minimal impact on GS accuracy leading to a greater return on investment from genotyping (Abed et al. 2018). Sequencing of multiplex PCR-based amplicons to capture high value SNPs may be an ideal low-density genotyping platform for GS applications, featuring low cost, robustness and scalability. Amplicon sequencing technologies assay a polymorphic panel of hundreds to a few thousand target SNP markers at the population scale with demonstrated applicability in phylogenetics (Dupuis et al. 2018), structure analysis (Andrews et al. 2016), and QTL mapping (Onda et al. 2018). Moreover, targeted amplicon sequencing effectively allows genotyping of small amounts of low quality DNA, even derived from dried herbarium samples (Beck and Semple 2015; Csernak et al. 2017). Therefore high throughput in-field sampling with minimal considerations on remote field stations is suitable. Amplicon panels are available for both the Illumina (Csernak et al. 2017) and the Ion torrent (Glotov et al. 2015) platforms and aim at interrogating allelic diversity at known loci of interest. The vast amount of genomic information available in rice, including a high quality reference genome (Matsumoto et al. 2005; Kawahara et al. 2013), 3010 re-sequenced varieties (Wang et al. 2018), de novo assemblies’ within different rice subpopulations (Schatz et al. 2014; Duitama et al. 2015) and high-density genotypes of diversity panels (Huang et al. 2010; McCouch et al. 2016), constitutes an ideal resource to accurately design custom amplicon panels with carefully selected, highly informative SNPs uniformly distributed across the rice genome and tailored for specific breeding applications.

Here, we report the development of a 1000-SNP (1 K) Rice Custom Amplicon assay, or 1k-RiCA, for the cost-effective amplification and sequencing of a thousand highly informative SNP sites in a 384-plex protocol that has wide applicability, good repeatability, high accuracy and high efficiency in genotyping breeding lines and populations derived from the *indica* subspecies of *Oryza sativa*. Furthermore we demonstrate applicability of 1k-RiCA for *indica* diversity studies, and genomic prediction for *indica* based breeding programs.

## Materials and methods

### Plant materials

Rice accessions genotyped with the 1k-RiCA and used for analyses in this study are listed in Additional file 1: S1. A set of 700 samples from a panel of 283 diverse inbred *Oryza sativa* rice lines replicated at different levels was used to estimate the markers call rate, heterozygosity, repeatability, concordance properties. Among the 283 diverse accessions, 185 have been classified in different subpopulations according to structure analysis performed by Wang et al. (2018) and McCouch et al. (2016), and specific breeding germplasm source (IRRI irrigated indica breeding program) with 150 known *indica* lines, that grouped the subpopulations *ind*, *ind1A*, *ind1B*, *ind2*, *ind3*, and *indx*, 24 *japonica*, grouping the subpopulations *trj, tej*, *temp*, *trop*, and *japx*, 8 *aus*, 2 *aromatic* (subpopulation *aro*) and 1 *admix*. The remaining 98 unclassified lines came from a panel of 70 pigmented ‘black rice’ lines, and 28 were advanced lines from different rice breeding programs. Consensus genotypes generated for the 283 accessions were used for principal component (PCA). An additional PCA analysis solely on *O. sativa* sp. *indica* lines was done with 177 diverse *indica* rice accessions, 41 *indica* ‘black rice’ accessions and 213 elite recombinant inbred lines (RILs) derived from 11 elite-by-elite *indica* x *indica* bi-parental populations from the International Rice Research Institute (IRRI) Favorable Environments Breeding Program (FEBP). A set of 57 F_1_ plants from six families derived from *indica* × *indica*, and *indica* × *japonica* crosses were used to test the accuracy of the 1k-RiCA to call heterozygous genotypes. The value of the 1k-RiCA for Genomic Selection (GS) was tested using 353 *indica* elite breeding lines derived from 30 elite-by-elite *indica* × *indica* bi-parental populations from IRRI’s FEBP in a series of cross-validation experiments.

### Design of the 1k-RiCA SNP assay

The 1K Rice Custom Amplicon assay or 1k-RiCA was designed on Illumina’s TruSeq Custom Amplicon (TSCA) 384 Index Kit technology (https://www.illumina.com) using Illumina’s proprietary workflow. Initially 1,554 genome-wide SNPs and 28 markers associated with highly valuable traits were provided to Illumina for an in-silico testing. Of the original 1,582 SNPs supplied to Illumina, 967 uniformly distributed genome-wide and 28 trait markers were retained in the 1k-RiCA after the iterative TruSeq custom amplicon design and validation process. The 967 genome-wide SNPs were selected from two publically available resources, the Cornell_6K_Array_Infinium_Rice or C6AIR chip (Thomson et al. 2017) and the 3,000 rice genomes (Alexandrov et al. 2015; Mansueto et al. 2017; Wang et al. 2018). The 604 markers from the C6AIR data set were selected based on their high call rates (> 95%) and high minor allele frequencies (MAF ≥ 0.4) determined from genotypic data available on 1,172 IRRI *indica* rice breeding lines and *indica* released varieties genotyped with the C6AIR. The remaining 363 markers from the 3,000 rice genomes were selected to fill physical distance gaps not captured by the C6AIR’s SNPs and filtered for high call rates (> 95%) and high minor allele frequencies (MAF ≥ 0.4) estimated across 1,174 *indica* landrace accessions and cultivated *indica* varieties sequenced within the 3,000 rice genomes dataset (Alexandrov et al. 2015; Mansueto et al. 2017, and Wang et al. 2018).

The 28 trait-related markers linked to 11 different important trait associated genes/QTLs were obtained from functional markers or markers demonstrated to be linked and associated with the respective gene/QTL as reported in the literature. These markers include one marker for gelatinization temperature (GT); *starch synthase IIa* or *alk* (Gao 2003, and Bao et al. 2006), three for apparent amylose content (AAC) associated with the *Waxy* gene alleles *wx*, *Wx*^*t*^, *Wx*^*g1*^, *Wx*^*g2*^, and *Wx*^*g3*^ (Dobo et al. 2010; Teng et al. 2017), one for the grain size locus *GS3* (Takano-Kai et al. 2009), one for rice tungro spherical virus (*rtsv1*) (Lee et al. 2010), 19 for bacterial leaf blight, BLB resistance genes; *xa5* (Iyer and McCouch 2004; Dilla-Ermita et al. 2017), *Xa7* (Romer et al. 2009; Dilla-Ermita et al. 2017), *xa13* (Chu et al. 2006), *Xa21* (Peng et al. 2015), *Xa4* (Li et al. 2001), and *Xa23* (Wang et al. 2015), and three for submergence tolerance; *Sub1A* (Septiningsih et al. 2009).

### Genotyping and SNP calling

Genomic DNA (gDNA) was extracted from leaf tissue of single plants using methodologies described in Dilla-Ermita et al. (2017) and based on either using CTAB (Murray and Thompson 1980), or KingFisher SBEadex kits (https://www.thermofisher.com). DNA quality was checked visually on 1% agarose gel, while DNA quantity was assessed using PicoGreen® (https://www.biotek.com), and Qubit 2.0 (https://www.thermofisher.com) fluorometric kits. The concentration of DNA was adjusted to be close to 10 ng/μL for library preparation. 384-plex indexing and pooling was performed as instructed by the manufacturer (https://www.illumina.com). Sequencing was performed using the MiSeq Sequencing-by-Synthesis Technology System as specified by illumina® (https://www.illumina.com). A custom SNP-calling pipeline described in the Additional file 1: S2, was used to assign variants on the 1k-RiCA amplicons through alignment to the Nipponbare rice genome MSU7 version (Kawahara et al. 2013). Final SNP data were merged with SNP map information and encoded with the physical position and chromosome number of the SNP markers in a Hapmap format (International T, Consortium H 2003).

### SNP filtering, repeatability, concordance and imputation

SNPs were removed if minor allele frequency (MAF) ≤ 0.01; heterozygous calls ≥10%; and call rate (CR) ≤ 75% using customs scripts written in R version 3.5.0 (R Core Team 2018) and deposited in Github (https://github.com/jdavelez/1k-RiCA-geno-filters/blob/master/jdavelez_1k-RiCA.R). For each SNP, heterozygosity was determined as the proportion of heterozygous calls among all successfully called genotypes. SNP call rate was defined as the proportion of successfully called genotypes among all samples used in the study. Repeatability, or the degree of consistent genotype calls between independent samples from the same accession, was calculated among 38 different accessions that had 4 or more independent replicates as *R* = 100 − *e*_*l*_, where 100 is the maximum value expressed in percentage of consistent genotype calls between independent replicates, minus the *mean error rate per locus* or *e*_*l*_ described by Pompanon et al. (2005) and measured as the ratio between the number of single-locus genotypes with at least one allelic mismatch (*m*_*l*_) and the number of replicated single-locus genotyped (*n*_*l*_) compared to a reference genotype (*e*_*l*_ = *m*_*l*_/*n*_*l*_), averaged across all replicated accessions. Concordance rate or the degree of consistent genotype calls from common SNPs assayed in two different genotyping platforms for the same accession was measured as the proportion of exact matched genotypes between common SNPs genotyped using two different genotypic platforms, 1k-RiCA versus C6AIR or 1k-RiCA versus 3,000 genomes, in the same accessions. For further GS analysis, the SNP filtered data was imputed in TASSEL v5.0 (http://www.maizegenetics.net/tassel) (Bradbury et al. 2007) using the LD KNNi imputation methodology with a High LD Sites 30 and Number of nearest neighbors of 30 using a LinkImpute algorithm (Money et al. 2015).

### Hierarchical clustering and principal component analysis

A hierarchical clustering analysis using Ward’s minimum variance method (Sokal and Michener 1958; Murtagh and Legendre 2014) was done using the R version 3.5.0 function ‘*hclust*’ (Murtagh and Legendre 2014; R Core Team 2018) where Ward’s clustering criteria is implemented and the dissimilarities are squared before cluster updating. A dendogram graph was built in R using the function ‘*plot (asphylo())*’ (R Core Team 2018). A principal component analysis (PCA) (Pearson 1901) was performed and visualized using the R function ‘*prcomp*’ (Mardia et al. 1979, and R Core Team 2018). The numbers of optimal clusters (*k*-means) observed in the PCA analysis was determined using the Silhouette method (Rousseeuw 1987) using the R function ‘*silhouette*’ from the R package ‘*cluster*’ (Maechler et al. 2013).

### Trait markers quality control evaluation

The ability of the 1k-RiCA trait markers to correctly identify the samples with the desired and undesired alleles was determined using the SNP Quality Control methods and variables described by Platten et al. (2019). The variables used were: i) ‘Utility’ described by Platten et al. (2019) as the “proportion (percentage) of a prospective breeding pool across which a marker could be used to introgress a QTL. This is equivalent to the proportion of the pool which does NOT carry the donor allele of a marker”, calculated as: #*cultivars withOUT favorable allele*/*Total* # *cultivars assesed*, ii) ‘False Positive Rate’ (‘FPR’), or “the proportion known negative genotypes incorrectly classified as having the target QTL allele. Assayed as the number of known recipients identified as not having an unfavorable allele of the marker (and thus incorrectly classified as having the target QTL allele)”, calculated as: #*recipients withOUT unfavorable allele*/*Total* # *recipients*, and iii) ‘False Negative Rate’ (‘FNR’) or “the converse of FPR, the proportion of known target QTL genotypes incorrectly classified as not having the desired QTL allele due to not having a favorable allele of the marker”, calculated as: #*donor withOUT favorable allele*/*Total* # *donors*. Utility, FPR and FNR were measured and analyzed for each individual trait marker and/or trait haplotypes for those traits with more than one molecular marker associated with them.

### F_1_-heterozygotes SNP calling concordance

To test the utility and accuracy of the 1k-RiCA to correctly called heterozygous genotypes, a set of 57 F_1_ plants derived from six different bi-parental crosses; 8 from IRRI 154 / A69–1 (*indica / japonica*, with 441 polymorphic SNPs), 14 from A-69-1 / IR 4630-22-2-5-1-3 (*japonica / indica*, with 280 polymorphic SNPs), 19 from IR 4630-22-2-5-1-3 / CSR 28 (*indica / indica,* with 105 polymorphic markers), 4 from CSR 28 / MANAW THUKHA (*indica / japonica*, with 342 polymorphic SNPs), 10 from MANAW THUKHA / IRRI 154 (*japonica / indica*, with 385 polymorphic SNPs), and 2 from MS11 / A69–1 (*japonica / japonica*, with 395 polymorphic SNPs) were genotyped along with their parents. For each bi-parental cross, a ‘predicted F_1_-genotype’ was generated by combining the SNPs haplotype from each homozygous parent into a genotypic profile of a pseudo-F_1_ plant. The ‘predicted F_1_-genotype’ was compared with the 1k-RiCA genotypes of each F_1_ plant and SNP concordances were estimated by calculating the percentage of exact genotypic calls that are similar between the ‘predicted’ and empirical F_1_ genotypes.

### Genomic selection

Three hundred fifty-three elite *indica* breeding lines from IRRI’s Favorable environment Breeding Program (FEBP) and 6 different agronomical checks were selected for genotyping with 965 polymorphic markers from the1k-RiCA. These lines were derived from 30 different bi-parental families of sizes varying from 1 to 36 individuals, with an average of 12 plants per family. Phenotyping of these lines took place at IRRI’s - Los Baños experimental station during the 2017 wet-season (WS) and 2018 dry-season (DS), and PhilRice’s Nueva Ecija experimental station during 2017 WS. The lines were phenotyped using an augmented p-rep design (Williams et al. 2011) with a replication of 1.2, for total 430 plots evaluated on each yield trial. Plot sizes were of 6.48 m2 (6 rows × 27 hills) in Los Baños, and 5.4 m2 (5 rows × 27 hills) in Nueva Ecija.

The target traits evaluated were days to flowering (‘FLW’), grain yield (‘GY’), and plant height (‘PH’). FLW was recorded as the number of days after sowing, when 50% of the plants in the plot produced flowers. GY was estimated from a 3.12 to 5 m^2^ plot harvested and weighed and corrected for moisture content using the formula: $GY=\left(\frac{100- MC}{86}\right)\times \left(\frac{Grain\ Weight\ in\  gr}{3.12\ m2}\right)\times (0.01)$. From this sample the grain yield per hectare was calculated. PH was the actual measurement in cm from soil to the tip of the tallest panicle (International Rice Research Institute 1996).

Mixed linear models using the function *lmer()* contained in the R package ‘lme4’ (Bates et al. 2014) were used to estimate BLUEs (Best Linear Unbiased Estimate) and BLUPs (Best Linear Unbiased Predictor) for all traits. Restricted Maximum Likelihood Estimation or RMEL method was used to estimate the variance components by setting the argument RMEL = TRUE in the *lmer()* function. The model was fit according to: *Y*_*ijk*_ = *μ* + *g*_*i*_ + *t*_*j*_ + *r*(*t*)_*jk*_ + *e*_*ijk*_. Where *Y*_*ijk*_ is the phenotypic observation on genotype ***i***, in the trial *j* and in replicate *k*, *μ*, the overall mean, *g*_***i***_, the genotype effect, *t*_*j*_, the trial effect, *r*(*t*)_*jk*_, is the replicate within trial effect, and *e*_*ijk*_ the residual. To obtain BLUPs for the genotypes, except for the overall mean, all the effects were considered random. To obtain BLUEs for the genotypes, the overall mean and genotypes were considered as fixed effects. Adjusted means of accessions (BLUEs) were extracted for each trait to be used as phenotypes in the genomic prediction models.

Broad sense heritability of accession means, *H*^***2***^, was calculated for each trait using the formula of Hallauer et al. (2010) as follows: ${H}^2=\frac{\sigma_g^2}{\sigma_g^2+\frac{\sigma_{gy}^2}{t}+\frac{\sigma_e^2}{tr}}$. Where *t* represents the mean number of trials in which accessions were tested and *r*, the mean number of plots per accessions across trials. Genotypes were assumed independent and identical distributed for estimating *H*^***2***^. Variance components were estimated from a linear mixed model using ‘lmer4’ and the model *Y*_*ijk*_ = *μ* + *g*_*i*_ + *t*_*j*_ + *r*(*t*)_*jk*_ + *e*_*ijk*_, where *Y*_*ijk*_ is the phenotypic observation on genotype ***i***, in the trial *j* and in replicate *k*, *μ*, the overall mean, *g*_***i***_, the genotype effect, *t*_*j*_, the trial effect, *r*(*t*)_*jk*_, is the replicate within trial effect, and *e*_*ijk*_ the residual. The argument RMEL in the *lmer()* function was set TRUE to estimate the variance components.

For the cross-validation studies six different genomic selection models were used to estimate genomic estimated breeding values (GEBVs) including ridge regression (Endelman 2011) and five Bayesian models; BayesA (scaled-t), BayesB (gaussian mixture), BayesC (scaled-t mixture) (Meuwissen et al. 2001; Habier et al. 2011), Bayesian Lasso (BL) (Park and Casella 2008), and Reproducing Kernel Hilbert Spaces Regressions fitting the markers and pedigree relationships (RKHS G + A) as random effects (Pérez and de los Campos 2014). The model RKHS G + A implements a reproducing kernel Hilbert space (Wahba 1990) regression fitting two random effects, one representing a regression on pedigree, $a\sim N\left(0,A{\sigma}_a^2\right)$, where A is a pedigree-derived relationship matrix, and one representing a linear regression on markers, $g\sim N\left(0,G{\sigma}_{gu}^2\right)$ where, G is a marker-derived genomic relationship matrix. The ridge regression model was tested using the R package ‘rrBLUP’ (Endelman 2011). The Bayesian models were implemented using the R package ‘BGLR’ (Pérez and de los Campos 2014) and the default prior parameters described in Pérez and de los Campos (2014) with a thinning value of 5, and 12,000 iterations with the first 2000 iterations discarded as burn-in. Trace plots as described by Pérez et al. (2010) were used to visually check conversion for some models selected at random. The samples residual variance data given by BGLR outputs can be plotted using the following script; *plot (scan(“varE.dat”), type = “o”)*, where *“varE.dat”* is a vector of the residual variance. Additionally pedigree-BLUPs (Henderson 1975) using the pedigree relationship matrix were estimated to compare the performance of genomic selection models.

A 5-fold (*k* = 5) cross validation experiment using 4/5 of the 353 lines as the training set to predict the remaining 1/5 of the validation set was used. Each cross validation was repeated 10 times using 10 independent partitioning of the accessions into the training set and validation set. The presence of highly related individuals in the dataset could have the effect of artificially inflating prediction abilities if the closest individuals are randomly assigned to different folds, and one of those folds are used a training. To control for this possibility a stratified cross validation strategy was used when designing the different folds by sampling individuals randomly within families defined using the pedigree information of the lines. The accuracy of each cross validation experiment was computed as the mean value of the 10 Pearson correlations (Pearson 1901) between the observations and the cross-validated GEBVs, also known as the predictive ability (Heslot et al. 2012).

## Results

### 1k-RiCA SNP assay design

**Fig. 1:**
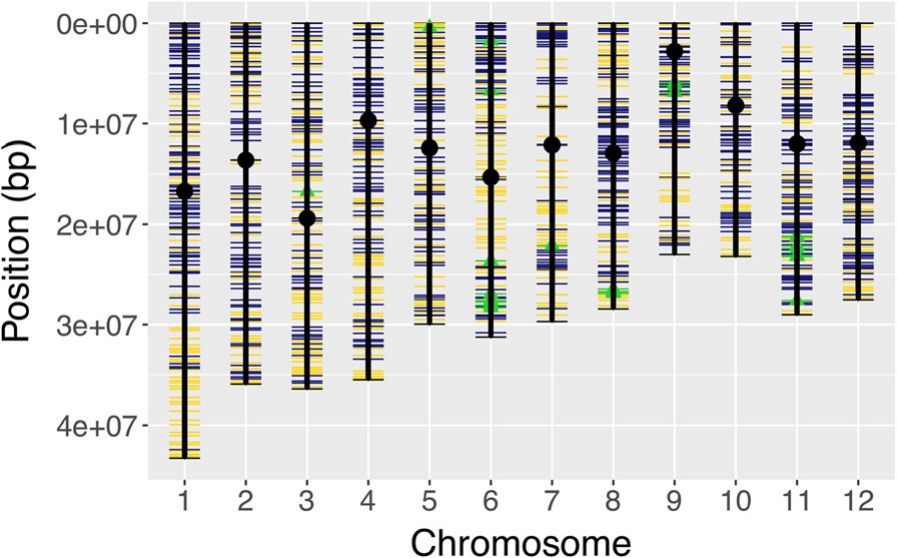
1k-RiCA SNPs physical positions. Genome-wide physical position distribution of 995 SNPs from the 1k-RiCA-assay across all rice chromosomes. SNPs designed from the C6AIR (Thomson et al. 2017) are represented in blue color, SNPs from the ‘3000 rice genomes’ are in yellow (Mansueto et al. 2017, and Wang et al. 2018), and trait-markers are in green

The 1k-RiCA was explicitly designed to be informative for *Oryza sativa* L*.* ssp. *indica* rice germplasm (see Materials And Methods). Out of the total 995 SNPs included and amplified in the 1k-RiCA, 604 markers were made up from the C6AIR (Thomson et al. 2017), 363 markers from the ‘3000 rice genomes’ (Mansueto et al. 2017, and Wang et al. 2018), and 28 markers linked to 11 different ‘high-valued’ trait genes/QTLs (Fig. 1 and Additional file 1: S3). Of the 995 SNPs, 482 markers localized within MSUv7 gene models (http://rice.plantbiology.msu.edu), and 513 were located within intergenic regions (Additional file 1: S3). The average physical distance between two adjacent markers across the whole genome in the 1k-RiCA set was 372 kb, or ~ 1.524 cM (SD = 1.2 cM), with 1 cM equal to ~ 244 kb (Chen et al. 2002). More than 50% of the markers are spaced from each other at a distance of 293 kb (~ 1.2 cM) or less (Additional file 2: Figure S1). The median SNP minor allele frequency (MAF) estimated from 1k-RiCA genotypic data on 431 *indica* accessions was 0.36 with 50% of the markers having MAF between 0.28 and 0.44 (Additional file 2: Figure S2).

The utility and accuracy of 28 markers associated with 11 different traits and designed for MAS strategies were evaluated using different Quality Control (QC) parameters described by Platten et al. (2019). Based on the results of the ‘Utility’, ‘False Positive Rates’ (‘FPR’), and ‘False Negative Rates’ (‘FNR’) QC parameter 21 SNPs were selected either to be used individually or as a haplotype for MAS for 11 different traits. A detailed description of the analysis results and the allelic interpretation for the selected SNPs is presented in Table 1. Individual SNPs for the traits associated with the loci *GS3*, *xa5*, *Xa4*, *Xa21*, *rstv*, and *ALK* are suitable for MAS. In addition, two or more markers associated with the loci *Xa7*, *xa13*, *Xa23*, *sub1*, and *Wx* for apparent amylose content (ACC) can be used as haplotypes for MAS applications (Table 1). More specifically, in the case of AAC, the haplotypes from the SNPs chr06:1.766, chr06:1.768, and chr06:1.769 can be used to differentiate high (G-A-C / G-A-T), intermediate (G-C-C), and low (T-A-C / T-C-C) ACC (Dobo et al. 2010).

**Table 1:** Quality Control assessment of 21 trait-SNPs contain in the 1K-RiCA assay. The QC parameters ‘Utility’, ‘False Positive Rate’ (FPR), ‘False Negative Rate’ (FNR) were estimated for each SNP and haplotypes when more than 1 SNP was associated with a trait

SNP ID	Chr.	MSU7 Pos.	Gene	Negative Allele	Positive Allele	Negative Trait	Positive Trait	Use as	SNP Utility	SNP FPR	SNP FNR	Haplotype Utility	Haplotype FPR	Haplotype FNR
chr03:16733441	3	16.73	*GS3*	G	T	Short	Long	Use as single SNP (T)	34.8%	0.0%	0.0%			
chr05:437499	5	0.44	*xa5*	T	A	Susceptible	Resistant	Use as single SNP (A)	91.4%	0.0%	0.0%			
chr06:27275515	6	27.28	*Xa7*	G	A	Susceptible	Resistant	Use haplotype (AAC)				96.2%	0.0%	0.0%
chr06:27627615	6	27.63	*Xa7*	C	A									
chr06:27761109	6	27.76	*Xa7*	A	C									
chr08:26448560	8	26.45	*xa13*	T	G	Susceptible	Resistant	Use haplotype (GGC)				81.8%	0.0%	0.0%
chr08:26709228	8	26.71	*xa13*	A	G									
chr08:26898822	8	26.90	*xa13*	T	C									
chr11:27603799	11	27.60	*Xa4*	C	A	Susceptible	Resistant	Use as single SNP (A)	52.4%	0.0%	0.0%			
chr11:21190115	11	21.19	*Xa21*	C	T	Susceptible	Resistant	Use as single SNP (T)	98.4%	0.0%	0.0%			
chr11:22162729	11	22.16	*Xa23*	C	T	Susceptible	Resistant	Use as haplotype (TTA)				96.5%	0.0%	0.0%
chr11:22453819	11	22.45	*Xa23*	C	T									
chr11:23231455	11	23.23	*Xa23*	G	A									
chr07:22119347	7	22.12	*rstv*	A	G	Susceptible	Resistant	Use as single SNP (G)	91.9%	0.0%	80.0%			
chr09:5922125	9	5.92	*sub1*	C	T	Susceptible	Tolerant	Use haplotype (TAG)				88.1%	0.0%	0.0%
chr09:6252407	9	6.25	*sub1*	G	A									
chr09:6913547	9	6.91	*sub1*	C	G									
chr06:6752756	6	6.75	*ALK*	A	G	Low-GT	High-GT	Use as single SNP	98.8%	0.0%	60.0%			
chr06:1765761	6	1.766	*Wxt/wx*	G	T	Depends on market preference	Depends on market preference	Use haplotype to differentiate high (GAC/GAC), intermediate (GCC) and low (TAC/TCC) ACC	84.3%	0.0%	0.0%			
chr06:1768006	6	1.768	*Wxin* *(Wxg1)*	A	C				60.8%	0.0%	75.0%			
chr06:1768998	6	1.769	*Wxa* *(Wxg2)*	C	T				75.2%	66.7%	100.0%			

### SNP call, heterozygosity, repeatability and concordance rate of the 1k-RiCA

SNP call rates and heterozygosity were empirically determined in the 1k-RiCA using 700 independent DNA samples derived from 283 partially replicated rice accessions. The mean call rate across all SNPs was 95% (or 0.95) (Additional file 2: Figure S3), and the mean heterozygosity observed among SNPs was of 1.5% (or 0.015) (Additional file 2: Figure S4). After removing 97 SNPs with less than 75% call rates and 5 more SNPs with heterozygosity values higher than 10% a total of 895 SNPs were kept for subsequent analysis in this set of samples.

The 1k-RiCA marker repeatability was measured on 38 different accessions replicated 4 or more times (Additional file 2: Figure S5). The average SNP repeatability observed on the replicated accessions genotyped with the 1k-RiCA was of 99% (Additional file 2: Figure S6). A thorough look at the 1% average genotyping mismatches showed a bias on miscalled heterozygous based on the inbred nature of these lines, accounting for 0.7% of the observed 1% mismatches. After heterozygous calls were removed and imputed the mean repeatability increased to 99.7% (Additional file 2: Figure S6).

SNP concordance rate between the 1k-RiCA and Cornell’s C6AIR, and the 1k-RiCA with the ‘3000 rice genomes’ were estimated and averaged across overlapping SNPs in commonly genotyped samples. To compare the 1k-RiCA and the C6AIR platform calls, a set of 600 overlapping SNPs across 34 different accessions were genotyped with both platforms. Concordance values across samples ranged between 96.5 and 100% with an average of 99.3%. Concordance of 271 overlapping SNPs between 1k-RiCA and the ‘3000 rice genomes’ data set was assessed across 10 different accessions and ranged from 97.7 to 100% with a mean of 99.17%.

The SNP technical quality properties of the 1k-RiCA were not affected when two different DNA extraction protocols, a modified CTAB (Murray and Thompson 1980) and a King-Fisher Kit (http://www.thermofisher.com) were tested in this study. The average SNP concordance rate between samples extracted with CTAB and King-Fisher Kit was 99.51% (Additional file 2: Figure S7).

### Principal component analysis in *O. sativa*

**Fig. 2:**
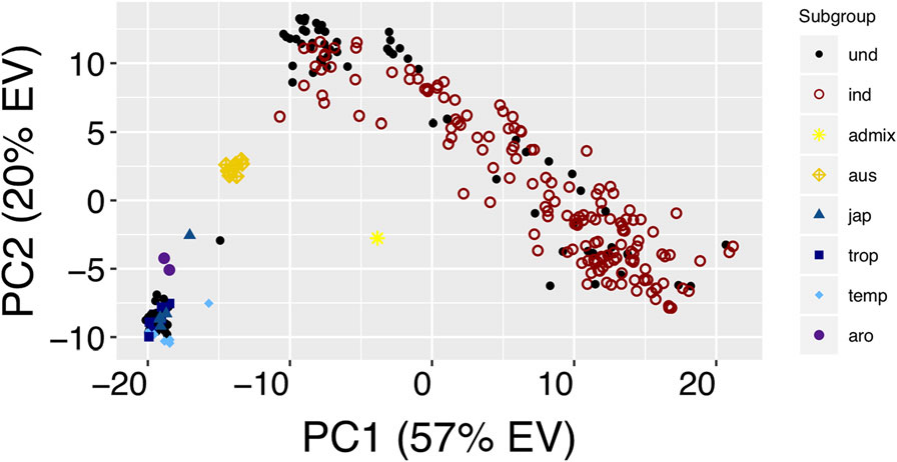
Principal component analysis of 283 *O. sativa* accessions using the 1k-RiCA genotypic data. Principal component analysis of 283 rice accessions genotyped with 895 SNP markers using the 1k-RiCA. Subpopulation classification for 185 accessions as *indica* (*ind*), *aus* (*aus*), *tropical japonica* (*trop*), *temperate japonica* (*temp*), *aromatic* (*aro*), and *japonica* (*jap*) was defined based of Wang et al. (2018) and McCouch et al. (2016) classification

A PCA using the 1k-RiCA was performed on 283 accessions, with 150 known *indica* lines (*ind*), 24 *japonica*, 8 *aus*, 2 *aromatic* (*aro*), 1 *admixture* (*admix*), and 98 undetermined (*und*) rice lines. The first principal component (PC1) explained 57% of the total genetic variation and separated the *indica*, *aus*, and the *japonica* varietals (*jap*, *temp*, *trop* and *aro*) accessions (Fig. 2). The second PC (PC2) explained ~ 20% of the total genetic variation and differentiated the *aus* from the *japonica* varietals. In addition, PC1 and PC2 captured a great portion of the variation within the *indica* accessions (Fig. 2). The optimal number of clusters estimated using Silhouette method identified 3 groups based on the genetic variance explained by the 1k-RiCA (Additional file 2: Figure S8A). One group contained the *Japonica* and *aromatic* lines (*jap*, *temp*, *trop,* and *aro*), the second group clustered *aus*, and *indica* lines (most of *indica* landraces, and *indica* “black rice”) and the final group contained most of the released and elite *indica* lines (Additional file 2: Figure S8B).

The undetermined accessions (*und*) accessions were then classified into two main groups ‘*Japonicas*’ (*jap*, *temp*, *trop*), and ‘*Indicas*’ (*ind*) using the PC coordinates from the PCA (Additional file 2: Figure S9). A manual cross-reference search on 12 different ‘*und*’ lines using publicly available data confirmed the predicted group based on the PCA with their reported subpopulation (Additional file 2: Table S1). Among the ‘*und*’ accessions a set of 78 lines part of a black-pericarp diversity panel was classified with 41 of them grouped as ‘*Indicas’*, and 37 as ‘*Japonicas’* (Additional file 2: Figure S9). Black dots ('und' - undetermined), open maroon circles ('ind' - *indica*), yellow stars ('admix' - admixture), ochre diamonds ('aus' - *aus*), blue trianges ('jap' - *japonica*), dark blue squares ('trop' - *tropical japonica*), light blue diamonds (temp - *temperate japonica*), purple dots ('aro' - *aromatic*).

### Principal component analysis within *indica* lines

**Fig. 3:**
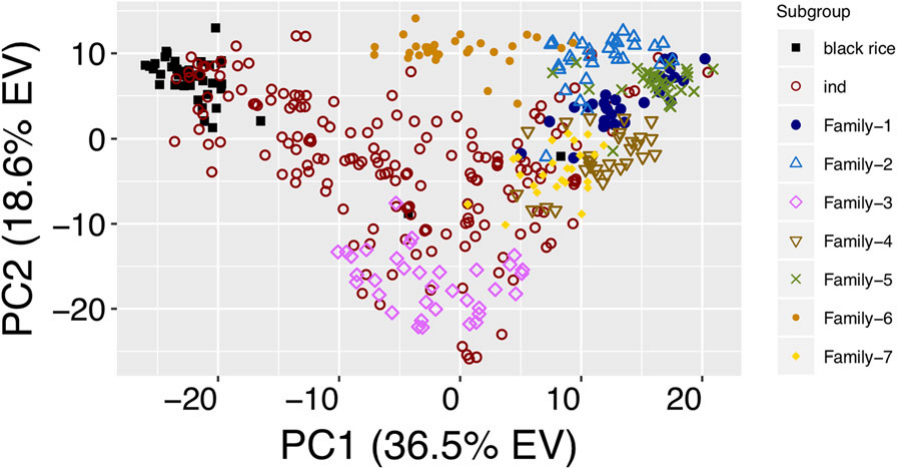
Principal component analysis of 431 *O. sativa S. indica* accessions using the 1k-RiCA genotypic data. PCA on 431 *‘Indica’* accessions including 177 diverse lines (*ind*), 41 ‘black rice’ (black rice), and 213 lines derived from 7 bi-parental families from IRRI’s Favorable Environments Breeding Program (Family). Diverse lines were color and coded as open maroon circles ('ind' - *indica*), as solid black squares ('black rice'). Elite breeding lines were classified by family (‘Family’) based on their pedigree data. Blue dots ('Family-1'), open blue triangles ('Family-2'), open magenta diamonds ('Family-3'), open inverted brown triangles ('Family-4'), green crosses ('Family-5'), ochre dots ('Family-6') and yellow diamonds ('Family-7')

To further determine the ability of the 1k-RiCA to assess the diversity within ‘*Indicas’*, a PCA was performed using 431 *‘Indica’* samples consisting of 177 diverse accessions, 41 ‘black rice’ accessions classified as *‘Indica’*, and 213 lines derived from seven different bi-parental families developed from crosses between elite *‘Indica’* rice lines (Fig. 3). The *‘Indica’* accessions were well distributed across the first and second principal components (Fig. 3). The ‘black rice’ samples were clearly separated from the bi-parental families in the first PC that explained 36% total genetic variance. ‘Black rice’ accessions grouped exclusively in the upper left corner of the PC1 vs. PC2 scatter plot. This cluster was mainly composed of *‘Indica’* landraces (Additional file 2: Table S2) while the bi-parental populations clustered in the opposite side of the first PC where most of the modern IRRI breeding lines were located (Fig. 3, and Additional file 2: Table S2). Within the bi-parental families the 1k-RiCA further distinguished the structure between the half-sibs families Family-1, Family-4, Family-5 and Family-7 that grouped closer to each other than the other families Family-3, and Family-6 (Fig. 3).

### Polymorphism rates across pairwise combinations

**Fig. 4:**
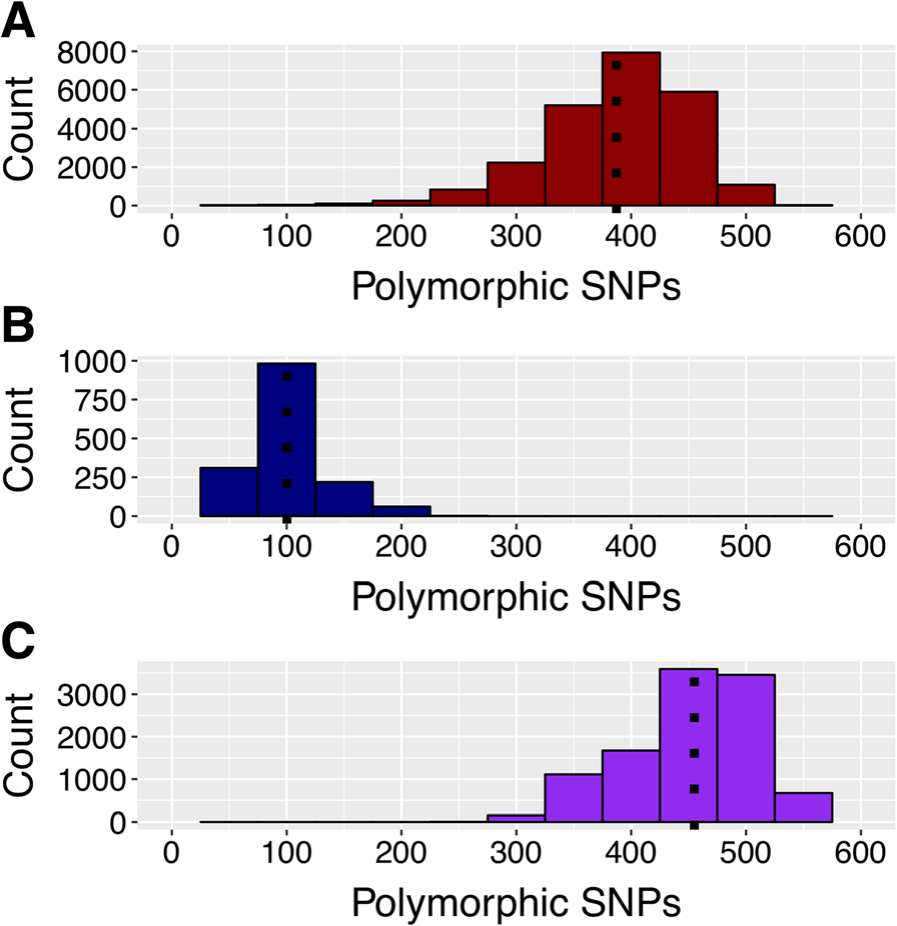
Polymorphic SNPs distribution across pairwise combinations of **a**) *indica x indica*, *japonica x japonica* **b**), and **c**) *indica x japonica.* Distribution of polymorphic markers between pairs of accessions from **a**) *indica* by *indica* (*ind* by *ind*), **b**) *japonica* by *japonica* (*jap* by *jap*), and **c**) *indica* by *japonica* (*ind* by *jap*) using 218 ‘*indica*’and 57 ‘*japonicas*’ accessions. The average number of polymorphic markers for each class combination is determined by a dotted line

Genotypic data generated from 275 accessions genotyped with the 1k-RiCA representing the two main rice sub-groups employed in rice breeding programs, ‘*Indica*’ (177 diverse lines and 41 ‘black rice’ *‘Indica’* accessions) and ‘*Japonica*’ (57 accessions), were used in pairwise comparisons to assess the number of polymorphic SNPs within each group and across groups. Within the 218 *‘Indica’* accessions the mean number of polymorphic SNPs across all pairwise combinations was 395 with 50% of the values ranging between 354 and 433 polymorphic SNPs (Fig. 4A). The average median gap between two SNP pairs across all possible cross combinations between the *‘Indica’* accessions was 0.542 Mbp ranging between 0.01 Mbp to 15.55 Mbp. Within the ‘*Japonica’* group the median number of polymorphic SNPs was 97 (Fig. 4B) with 50% of the values ranging from 79 to 114. The average median gap between the *‘Japonica’* accessions was 1.61 Mbp ranging from 0.08 Mbp to 25.68 Mbp.

When pairwise combinations were estimated for interspecific crosses between *‘Indica’* and *‘Japonica’* accessions the median number of polymorphic SNPs observed was 465 (Fig. 4C). The average median gap between two SNP pairs across all possible cross combinations between the *‘Indica’* × *‘Japonica’* accessions was 0.5 Mbp (0.004–13.08 Mbp).

### F_1_ genotypes

For each bi-parental family, F_1_-plant genotypes were compared to the ‘predicted-F_1_’ progeny genotype. Across the 55 F_1_ plants the average percentage of similarity with the ‘predicted-F_1_’ was 99.22%, ranging from 97 to 99.87% (Additional file 2: Figure S10).

### Genomic selection

**Fig. 5:**
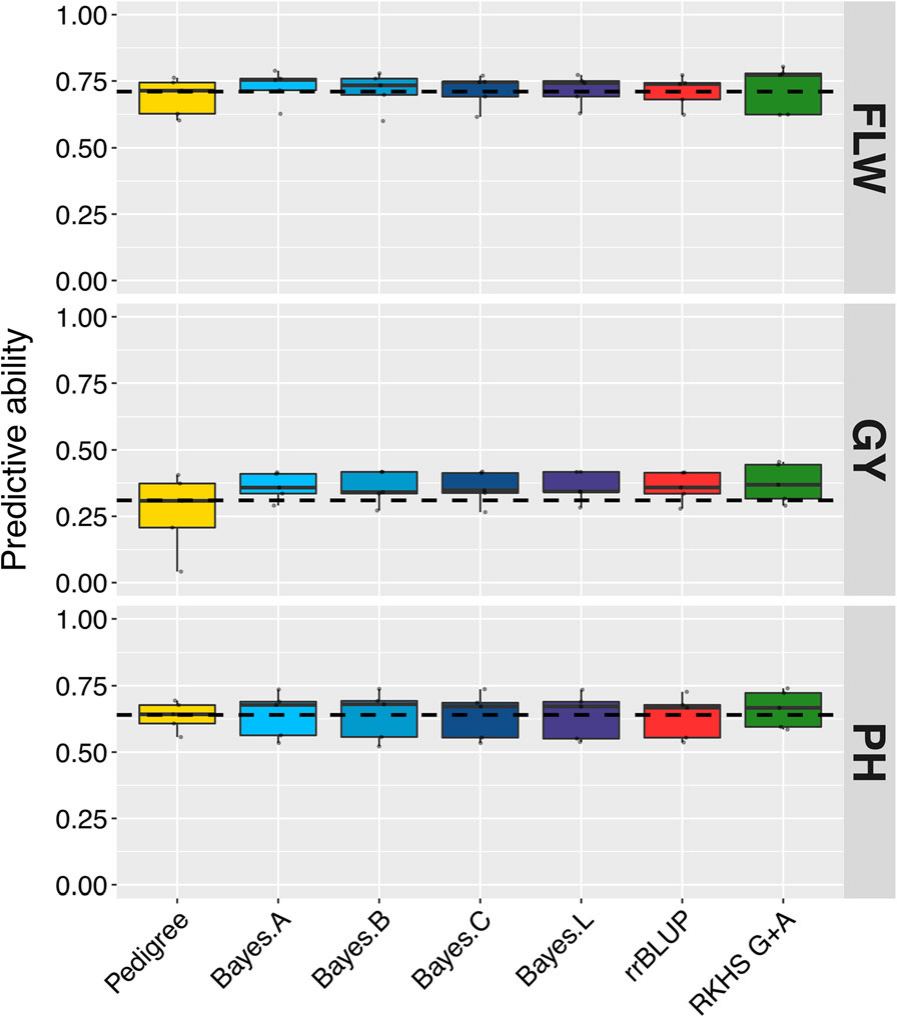
Prediction abilities of genomic selection models for FLW, GY and PH based on 5 fold cross-validation using the 1k-RiCA. Average predictive abilities across 5 fold stratified cross validations experiments (*k* = 5) using 353 rice lines measured for flowering time (FLW), grain yield (GY), and plant height (PH) for seven different statistical models; Pedigree BLUP (Pedigree), BayesA, BayesB, BayesC, BayesLasso (Bayes L), rrBLUP (ridge regression) and RKHS using genomic and pedigree relationship matrices (RKHS G + A)

Phenotypic data on the three traits used for cross validation of GS models, flowering time (FLW), grain yield (GY), and plant height (PH), exhibited a Gaussian distribution (Additional file 2: Figure S11). Broad sense heritability of accessions means, *H*^*2*^, was higher for PH (0.8), follow by FLW (0.85), while GY (0.5) had the lowest *H*^*2*^. Average selection predictive ability across 21 cross validation studies involving six different genomic selection models and one pedigree BLUP model on three traits were estimated. The predictive ability across all eight models ranged from 0.69 to 0.73 for FLW, from 0.27 to 0.38 for GY, and from 0.63 to 0.66 for PH. The genomic selection model RKHS that included the marker derived genomic and the pedigree derived relationship matrixes (G + A) had the highest predictive ability for GY and PH, while Bayes. A had the highest predictive ability for FLW (Fig. 5). The pedigree BLUP estimation method had the lowest predictive ability for FLW and GY while ridge regression had the lowest predictive ability for PH (Fig. 5).

## Discussion

The 995 SNPs on the final 1k-RiCA assay are uniformly distributed across the rice genome. The physical length of the Nipponbare reference genome is 373,245, 519 bp (http://rice.plantbiology.msu.edu) or 1,529.7 cM, with 1 cM equivalent to 244 kb (Chen et al. 2002). If 995 markers were uniformly distributed across all chromosomes they would be on average 1.53 cM apart. This value does not differ from the empirical estimations for the 1k-RiCA with a mean distance of 1.524 cM between adjacent markers. Evident gaps, or regions without a marker were found in the centromeric region of chromosomes 2 and 7, the long arm of chromosomes 6 and 9, as well as the telomeric regions of chromosomes 11 and 12. These few regions where markers are less uniformly distributed could be addressed in the future since this technology allows the introduction of additional target loci when new kits are designed. Uniformly distributed SNP sets have been reported to be useful for breeding applications to develop interspecific populations (Orjuela et al. 2010), conduct QTL analysis, characterize the genetic structure of rice populations (Thomson et al. 2017), and implement genomic selection (Habier et al. 2009). Equidistant distribution maximizes detection ability of recombination events with the given marker density and minimizes distance to causal trait-contributing polymorphisms for QTL detection.

As opposed to classical genotyping-by-sequencing (GBS) approaches (Elshire et al. 2011) the 1k-RiCA consistently scores the same set of SNPs with SNP call rates average of 95%, resulting in identical genotype matrixes, which facilitates analyses across multiple runs without further bioinformatics. While array based technologies provide similar consistencies at higher densities, they are significantly more expensive to run at a significantly lower throughput making the 1k-RiCA more suitable for high throughput breeding applications that rely on fast turnaround time for decision making and benefit from a lower per-sample cost at the sacrifice of SNP density.

Markers from the 1k-RiCA showed high levels of repeatability across independently genotyped samples (> 99%) and high concordance rates with the respective SNP alleles reported for the same accession within the C6AIR and 3000 rice genomes datasets. The 1% genotypic mismatches observed in the repeatability analysis was to a large portion due to miscalled heterozygous loci in inbred lines and through imputation this source of error was reduced to 0.3% increasing repeatability to 99.7%. Comparison of SNP calls between 1k-RiCA and two different platforms, C6AIR and 3000 rice genomes showed on average values of 99% accuracy. These empirical results demonstrated the high levels of repeatability, accuracy, and robustness of the 1k-RiCA, which are comparable to those reported in TruSeq Amplicon panels for cancer clinical testing (Simen et al. 2015; Misyura et al. 2016), and other genotyping platforms, such as the C6AIR in rice (Thomson et al. 2017), and the CottonSNP80K in cotton (Cai et al. 2017).

Quality of the 1k-RiCA did not differ when two different DNA extraction protocols were compared. The cheap and ‘quick and dirty’ manual CTAB extraction method and semi-automated magnetic bead-based DNA extraction and purification yielded similar results, giving the 1k-RiCA an additional advantage over reduced-representation sequencing genotyping approaches which necessitate the use of specific restriction enzymes, that tend to be sensitive to contaminants often carried over by precipitation-based extraction methods such as CTAB. The highly purified DNA preparations using column- or magnetic-based systems required for reduced-representation sequencing genotyping approaches, however, are significantly more expensive to obtain and often not available to resource-limited breeding programs.

The 1k-RiCA adequately captured the diversity present in *‘Indica’* accessions. It was able to differentiate within and between *‘Indica’* landraces and breeding populations (Fig. 3). It did not, however, adequately differentiate between and within the *aromatic*, *temperate*, and *tropical-japonica* subgroups (Fig. 2).

These limitations stem from the original design and purpose of this platform, which was to capture the diversity of *‘Indica’* lines of tropical rice breeding programs across South Asia and South East Asia, including IRRI’s FEPB. By filtering for high MAF within the *‘Indica’* subgroup, many of the selected SNPs displayed low MAF or even monomorphism within *‘Japonica’* subgroups and hence did not contribute to power of discrimination. The low average number of polymorphic rates between *‘Japonica’* and *‘Japonica’* comparisons (97) makes the 1k-RiCA unsuitable for *temperate* and *tropical japonica* breeding programs or diversity analyses. On the other hand the 1k-RiCA showed similar average numbers of polymorphic SNPs in pairwise combinations between *‘Indica’* and *‘Indica’* (395) and between *‘Indica’* and *‘Japonica’* (465) (Fig. 4), making it suitable for the analysis of intra-specific breeding populations and inter-specific populations between *‘Indica’* and *‘Japonica’* accessions.

The genotyping results of the F_1_ *indica* × *indica*, and *indica* × *japonica* plants showed the 1k-RiCA accurately called heterozygous genotypes. Considering that the mean repeatability estimated in the study is about 99%, the 1% of dissimilarities between predicted and F_1_ calls can be explained by genotypic errors of the 1k-RiCA. The ability to robustly call heterozygous genotypes makes the 1k-RiCA suitable for genotyping segregating populations, marker assisted backcrossing (MABC) and genomic prediction in segregating populations.

A major challenge in implementing GS in public rice programs is the cost associated with genotyping. The expected value of the information gained by genotyping must exceed the cost of obtaining genotype information (Boichard et al. 2012). The most straightforward approach to reduce per-sample genotyping cost is by reducing SNP density and increasing multi-plexing of samples per NGS run to a point that does not jeopardize prediction accuracies.

Testing the 1k-RiCA data as genotypic input for genomic prediction in 21 cross-validation experiments using six different GS, and one pedigree model demonstrated its suitability for predicting complex traits such as flowering time (FLW), grain yield (GY), and plant height (PH). The genomic selection prediction abilities for FLW (0.69–0.73), GY (0.27–0.38) and PH (0.63–0.66) were comparable to those reported by Spindel et al. (2015) for the same traits (FLW = 0.63, GY = 0.31, PH = 0.34), using 73,147 SNP markers, and rice materials from the same breeding program. Furthermore, the observation that the RKHS G + A model was more accurate than the pedigree BLUP model indicates that the markers are effective in capturing the variation among relatives due to Mendelian sampling, which is key for being able to select within families effectively based on prediction. Spindel et al. (2015) suggested that using ~ 1 SNP every 0.2 cM (~ 6 K SNPs) could be ideal for performing selection in inbred rice breeding populations. Grenier et al. (2015) estimated that in rice with a map of 18 Morgans, and effective population size (Ne) of ~ 50, about 3,600 SNPs would be needed under an infinitesimal model with additive effects and under the assumptions of evenly distributed QTLs on the chromosome for genomic prediction purposes. However, in their empirical confirmation study using a cross validation analysis in upland rice, the greatest accuracies were achieved with a matrix size of 1,700 SNPs, suggesting that the assumptions presented in the simulation study did not necessarily apply. Furthermore a GS optimization study in wheat has shown that 1,000 marker were enough to reach the highest predictive ability for GS in a breeding program (Cericola et al. 2017). Similar optimization results were also obtained for a GS study in barley where a minimum marker set of 1,000 was found to be necessary in order to decrease the risk of low prediction accuracies (Nielsen et al. 2016). The predictive abilities obtained in this study suggest that the marker density for the 1k-RiCA may be sufficient and currently cost-effective for the application of GS in elite rice *indica* germplasm.

Alternatively the integration of imputation in the 1k-RiCA could increase genomic prediction accuracies if high density genotypic information is generated from the parental material of GS tested population. Increases in prediction accuracies using this approach have been observed in simulation studies (Gorjanc et al. 2017) and empirical studies in cattle (Wang et al. 2016), salmon (Tsai et al. 2017), and rapeseed (Werner et al. 2018).

The introduction of 21 trait markers associated with 11 different traits of agronomic importance adds to the utility of the 1k-RiCA. While it would not be cost effective to run the 1k-RiCA solely for the trait information (single SNP assay chemistry such as KASP would be cheaper), it enables the application of marker assisted selection (MAS) and trait profiling in conjunction with fingerprinting. In breeding programs this facilitates the enrichment of favorable alleles, while in diversity-type analyses it allows for the assessment of presence/absence of a range of traits. While some of the markers are diagnostic and can be used individually others are only linked to the causal polymorphism and are best used in combination as haplotypes. The use of haplotypes adds to robustness, since single linked markers might not be predictive in unknown backgrounds, where linkage may be broken.

Apart rom direct use in MAS, the presence of these markers opens the possibility of refining genome wide prediction models. Using trait marker information as fixed effect parameters has the potential to increase selection accuracies as reported by Rutkoski et al. (2014) for adult plant resistance to stem rust in wheat, by Spindel et al. (2015) for rice plant height and by Lopes et al. (2017) in livestock. The 1k-RiCA can be used efficiently by combining these two different molecular breeding approaches for traits associated with bacterial leaf blight, grain physical and chemical quality traits, submergence tolerance and other biotic stresses.

## Additional files

Additional file 1: **S1.** List of accessions used in this study. **S2.** SNP call pipelined used to identify genotypes in the 1k-RiCA. **S3.** List of 995 SNPs contained in the 1k-RiCA. **S4.** Hap-map formatted 1k-RiCA genotypic data on 283 lines used in the *O. sativa* PCA. **S5.** Hap-map formatted 1k-RiCA genotypic data on 431 lines used in the *O. sativa* ssp. *indica* diversity analysis. **S6.** Hap-map formatted 1k-RiCA genotypic data on 57 F_1_ lines and their parents used in the F_1_ analysis. **S7**. Hap-map formatted 1k-RiCA genotypic data on 353 lines used in the genomic selection cross-validation experiments. **S8.** Adjusted phenotypic means for FLW, GY, and PH on 353 lines used in the genomic selection cross-validation experiments. (XLSX 8888 kb)

Additional file 2: **Figure S1.** Distribution of physical distance gaps between adjacent SNPs in the 1k-RiCA. **Figure S2.** 1k-RiCA SNP minor allele frequency (MAF) estimated on 431 *indica* lines. **Figure S3.** 1k-RiCA SNP call rate distribution. **Figure S4.** 1k-RiCA SNP heterozygosity distribution. **Figure S5.** Hierarchical cluster analysis estimated in 38 highly replicated accessions using the 1k-RiCA set. **Figure S6.** 1k-RiCA average SNP repeatability. **Figure S7.** 1k-RiCA SNP concordance rate distribution between CTAB and King-Fisher DNA extracted samples. **Figure S8.** 1k-RiCA *Oryza sativa* PCA optimal clustering and Silhouette width test. **Figure S9.** 1k-RiCA *Oryza sativa* PCA classification. **Figure S10.** 1k-RiCA distribution of F_1_ percentage of similarity between true F_1_ and predicted F_1_. **Figure S11.** Phenotypic distribution for flowering time (FLW), grain yield (GY), and plant height (PH). **Table S1.** Cross-reference identification for 12 ‘*undermined*’ rice accessions classified using PC coordinates. **Table S2.** Grouping analysis using PC coordinates between *indica* lines. (DOCX 925 kb)

## Data Availability

The datasets supporting the conclusions of this article are included within the article and its Additional file 1: S1, S2, S3, S4, S5, S6, S7 and S8 and Additional file 2.

## Abbreviations

1k-RiCA: 1K - Rice Custom Amplicon
AAC: Apparent amylose content
FEBP: Favorable Environments Breeding Program
FLW: Flowering
FNR: False Negative Rate
FPR: False Positive Rate
GBS: Genotyping-by-sequencing
gDNA: Genomic DNA
GEBV: Genomic estimated breeding values
GS: Genomic selection
GT: Gelatinization temperature
GY: Grain yield
IRRI: International Rice Research Institute’s
LOD: Logarithm of the odds
LRG-RRG: Longest root growth
MABC: Marker assisted backcrossing
MAF: Minor allele frequencies
MAS: Marker assisted selection
MERL: Mean error rate per locus
NGS: Next generation sequencing
NILs: Near isogenic lines
PGR-RRG: Primary root growth
PH: Plant height
PVE: Percentage phenotypic variation explained
PYs: Pyramided lines
QC: Quality Control
RILs: Recombinant inbred lines
RRS: Reduced representation sequencing
SNP: Single nucleotide polymorphism
TRG-RRG: Total root growth
TSCA: TruSeq Custom Amplicon
WS: Wet-season
