# Supplementary material for: 1k-RiCA (1K-Rice Custom Amplicon) a novel genotyping amplicon-based SNP assay for genetics and breeding applications in rice
Source: Rice (N Y). 2019 Jul 26;12:55. doi: 10.1186/s12284-019-0311-0 (PMC6660535; doi:10.1186/s12284-019-0311-0)
Supplement: Supplementary file 2 — Figure S1. Distribution of physical distance gaps between adjacent SNPs in the 1k-RiCA. Figure S2. 1k-RiCA SNP minor allele frequency (MAF) estimated on 431 indica lines. Figure S3. 1k-RiCA SNP call rate distribution. Figure S4. 1k-RiCA SNP heterozygosity distribution. Figure S5. Hierarchical cluster analysis estimated in 38 highly replicated accessions using the 1k-RiCA set. Figure S6. 1k-RiCA average SNP repeatability. Figure S7. 1k-RiCA SNP concordance rate distribution between CTAB and King-Fisher DNA extracted samples. Figure S8. 1k-RiCA Oryza sativa PCA optimal clustering and Silhouette width test. Figure S9. 1k-RiCA Oryza sativa PCA classification. Figure S10. 1k-RiCA distribution of F1 percentage of similarity between true F1 and predicted F1. Figure S11. Phenotypic distribution for flowering time (FLW), grain yield (GY), and plant height (PH). Table S1. Cross-reference identification for 12 ‘undermined’ rice accessions classified using PC coordinates. Table S2. Grouping analysis using PC coordinates between indica lines. (DOCX 925 kb) [file 12284_2019_311_MOESM2_ESM.docx]

**Additional file 2: Figures and Tables: 1k-RiCA (*1k-Rice Custom Amplicon*) a novel genotyping amplicon-base SNP assay for genetics and breeding applications in rice**

**Juan David Arbelaez**. International Rice Research Institute, Los Baños, Philippines, DAPO Box 7777 Metro Manila 1301.Email: j.arbelaezvelez@irri.org

**Maria Stefanie Dwiyanti**. Research Faculty of Agriculture, Hokkaido University, Sapporo, Hokkaido 060-8589, Japan. Email: dwiyanti@abs.agr.hokudai.ac.jp

**Erwin Tandayu**. International Rice Research Institute, Los Baños, Philippines, DAPO Box 7777 Metro Manila 1301.Email: e.tandayu@irri.org

**Krizzel Llantada**. International Rice Research Institute, Los Baños, Philippines, DAPO Box 7777 Metro Manila 1301.Email: k.llantada@irri.org

**Annalhea Jarana**. International Rice Research Institute, Los Baños, Philippines, DAPO Box 7777 Metro Manila 1301.Email: a.jarana@irri.org

**John Carlos Ignacio**. International Rice Research Institute, Los Baños, Philippines, DAPO Box 7777 Metro Manila 1301.Email: j.ignacio@irri.org

**John Damien Platten**. International Rice Research Institute, Los Baños, Philippines, DAPO Box 7777 Metro Manila 1301.Email: J.Platten@irri.org

**Joshua Cobb**. International Rice Research Institute, Los Baños, Philippines, DAPO Box 7777 Metro Manila 1301.Email: j.cobb@irri.org

**Jessica Elaine Rutkoski**. International Rice Research Institute, Los Baños, Philippines, DAPO Box 7777 Metro Manila 1301.Email: j.rutkoski@irri.org

**Mike Thomson**. Department of Soil and Crop Sciences, Texas A&M University, College Station, Houston, TX 77843, ISA. Email: m.thomson@tamu.edu

**Tobias Kretzschmar**. Southern Cross Plant Sciences, Southern Cross University, PO Box 157, Lismore NSW 2480, Australia. Tobias.Kretzschmar@scu.edu.au

**Figure S1. Distribution of physical distance gaps between adjacent SNPs in the 1k-RiCA.** SNP-distance distribution between adjacent SNPs in the 1k-RiCA expressed in a 1,000 bp (1 Kbp) scale. The mean SNP distance between adjacent SNPs, ~372 kb, is indicated by a dotted line.

**Figure S2. 1k-RiCA SNP minor allele frequency (MAF) estimated on 431 *indica* lines.** Distribution of the 1k-RiCA MAF on 995 SNPs using 431 *indica* inbred lines. The median SNP MAF value of 0.355 estimated is defined by a dotted line.

**Figure S3. 1k-RiCA SNP call rate distribution.** Distribution of the 1k-RiCA SNP call rate estimated on 995 SNPs using 700 independent DNA samples. The mean SNP CR value of 0.95 (or 95% CR) estimated is defined by a dotted line.

**Figure S4. 1k-RiCA SNP heterozygosity distribution.** Distribution of the 1k-RiCA SNP heterozygosity estimated on 995 SNPs using 700 independent DNA samples. The mean SNP heterozygosity value of 0.015 (or 1.5%) estimated is defined by a dotted line.

**Figure S5. Hierarchical cluster analysis estimated in 38 highly replicated accessions using the 1k-RiCA set.** Replicates were extracted using the Kingfisher kit and a modified CTAB protocol. Samples extracted with CTAB are names with the suffix “.ct.” after their ‘SW’ identity code. Labels with the same color belong to the replicates of the same accession.

**Figure S6. 1k-RiCA average SNP repeatability**. Distribution of repeatability estimated in 38 highly replicated accessions using the 1k-RiCA-assay. The SNP repeatability distributions before and after removing miscalled heterozygous calls are indicated in red and blue respectively. Their corresponding means are indicated dotted (0.99 or 99%) and dashed lines (0.997 or 99.7%).

**Figure S7. 1k-RiCA SNP concordance rate distribution between CTAB and King-Fisher DNA extracted samples.** Distribution of the 1k-RiCA SNP concordance rate estimated between same samples extracted using CTAB and King-Fisher Kit. The mean SNP SNP concordance rate value of 0.99.51 (or 99.51%) estimated is defined by a dotted line.

**A)**

**B)**

**Figure S8. 1k-RiCA *Oryza sativa* PCA optimal clustering and Silhouette width test**. **8A)** Optimal number of clusters visually determined by the ‘Average silhouette width. **8B)** Classification of 283 accessions implementing the PCA coordinates using clusters generated using k-mean of 3 estimated to be the optimal number of cluster using the Silhouette method.

**Figure S9. 1k-RiCA *Oryza sativa* PCA classification**. Classification of ‘*undetermined*’ accessions implementing the PCA coordinates using clusters generated using 65 accessions that are known to be from the subpopulation indica (*IND*), aus (*AUS*), and *japonicas* (made up from *temperate japonica*, *tropical japonica*, and *aromatic* - JAPONICA). ‘Black rice’ *indica* and *japonica* accessions were identified with the ‘brc’ prefix.

**Table S1. Cross-reference identification for 12 ‘*undetermined*’ rice accessions classified using PC coordinates**. Manual cross-reference of 12 ‘un-determined’ accessions classified into 3 subpopulations using the PC coordinates from the 1k-RiCA diversity analysis.

| **Accession** | **Subpopulation** | **Description** | **Reference** | **Predicted population** |
| --- | --- | --- | --- | --- |
| BORUBI::IRGC25181-1 | *tropical japonica* | Diversity | McCouch et al 2016, Wang et al. 2017 | *japonica* |
| BLACK GORA S.N 109 | *indica* | India | Yan et al. 2011 | *indica* |
| BASTMATI | *indica* | Fragrance | Kovach et al. 2009 | *indica* |
| DPRK 036S 474 | *temperate japonica* | DPRK | Kim et al. 2014 | *japonica* |
| DPRK 036M 10515 | *temperate japonica* | DPRK | Kim et al. 2014 | *japonica* |
| DPRK 036S 9917 | *temperate japonica* | DPRK | Kim et al. 2014 | *japonica* |
| DPRK 036S 7220 | *temperate japonica* | DPRK | Kim et al. 2014 | *japonica* |
| Hei Mi Chan | *indica* | NA | Li et al. 2004 | *indica* |
| PIKTO MAMAS | *tropical japonica* | NA | Alexandrov et al. 2017 | *japonica* |
| BR11 | *indica* | NA | Fujita et al. 2013 | *indica* |
| IR74 | *indica* | NA | Lepitan et al. 2007 | *indica* |
| BR29 | *indica* | NA | Datta 2006 | *indica* |

**Table S2. Grouping analysis using PC coordinates between *indica* lines**. Accessions from the *indica* subpopulation that cluster together with the ‘Black-rice’ lines genotyped with the 1k-RiCA.

| **Accession** | **Subgroup cluster** | **Designation** | **PC1** | **PC2** |
| --- | --- | --- | --- | --- |
| HB2120 | Black rice | KHAO DAWK MALI 105 | -22.4216236 | 7.796715943 |
| HB2137 | Black rice | SAMBHA MAHSURI | -16.90539817 | 4.06043655 |
| QMp03 | Black rice | Birain 360 | -21.05959218 | 8.465602172 |
| QMp07 | Black rice | Birain 360 | -21.26630577 | 8.439611855 |
| SW108 | Black rice | IR 84649-97-3-1-B-B | -20.87644391 | 4.853705157 |
| SW112 | Black rice | ADR52 | -18.50421528 | 10.85843889 |
| SW130 | Black rice | Tadukan | -18.38149871 | 8.532461147 |
| SW165 | Black rice | BR11 | -23.5933901 | -0.317880888 |
| SW191 | Black rice | A 69-1 | -19.8083939 | -0.385602509 |
| SW211 | Black rice | Ashi Binni | -19.74753357 | 9.05212594 |
| SW236 | Black rice | POKKALI | -22.36954214 | 7.159561487 |
| SW237 | Black rice | SADU CHO | -21.49011261 | 7.475529874 |
| SW241 | Black rice | ZHENSHAN 97 B | -19.1458832 | 4.956531651 |
| SW242 | Black rice | ASWINA | -19.8401169 | 7.347433687 |
| SW251 | Black rice | MANAW THUKHA | -21.64006458 | 3.400897709 |
| SW264 | Black rice | BR 29 | -20.5146175 | -3.904192842 |
| SW266 | Black rice | IR 64680-81-2-2-1-3 | -20.79642326 | -0.650956341 |
| SW282 | Black rice | TETEP | -17.45662357 | 8.842468136 |
| SW291 | Black rice | Utri Merah IRGC 16682 | -23.45072245 | 7.009314534 |
| SW314 | Black rice | BRRI DHAN54 | -21.57019704 | 1.107270377 |
| SW321 | Black rice | IR 66946-3R-178-1-1 | -16.11874696 | -1.916090656 |
| SW350 | Black rice | JUMBO JET | -18.06171717 | 5.712257661 |
| SW352 | Black rice | CAPSULE | -20.27054761 | 8.501741355 |
| SW361 | Black rice | NONA BOKRA | -21.66435158 | 8.746726403 |
| SW363 | Black rice | SUPA | -22.21844476 | 9.573561571 |
| SW371 | Black rice | TKM6 | -15.57743275 | 5.770844922 |
| SW42 | Black rice | IR05F102 | -21.24373309 | 2.821051612 |
| HB2040 | Bi-parental | IRRI175 | 0.412580145 | -12.65065717 |
| HB2041 | Bi-parental | IRRI179 | 6.702205952 | -4.035194311 |
| HB2042 | Bi-parental | IRRI180 | 9.61331785 | -0.770119311 |
| HB2110 | Bi-parental | IRRI 104 | 7.962845049 | 0.604329164 |
| QMp01 | Bi-parental | IR36ae | 4.988906779 | -11.21331514 |
| SW103 | Bi-parental | NSIC Rc 342SR | 0.45527379 | -9.951786327 |
| SW142 | Bi-parental | IR03A262 | 3.069949619 | -2.928799249 |
| SW145 | Bi-parental | IRRI 149 (NSIC Rc 194) | 0.177181319 | -24.43838657 |
| SW146 | Bi-parental | PSB Rc 82 (IRRI 123) | 9.329657705 | -4.961634307 |
| SW16 | Bi-parental | IR10N112 | 0.203893536 | -17.57353826 |
| SW161 | Bi-parental | IR09F153 | 0.831732777 | -4.062617899 |
| SW18 | Bi-parental | IR09F154 | 6.694568584 | 3.82427151 |
| SW184 | Bi-parental | MTU 1010 | 1.263401683 | -11.48499906 |
| SW20 | Bi-parental | IR09F437 (PSB-Rc18-SUB1) | 8.221538344 | 4.829853322 |
| SW213 | Bi-parental | NSIC Rc 358 | 4.00353612 | -6.027681405 |
| SW222 | Bi-parental | NSIC Rc 308 | 3.553328343 | -13.94724575 |
| SW229 | Bi-parental | IR64-21 | 0.747691202 | -25.83314203 |
| SW24 | Bi-parental | IR74 | 1.021090933 | -0.940684958 |
| SW244 | Bi-parental | IR42 | 0.973807238 | -4.520028958 |
| SW250 | Bi-parental | IR11A293 | 13.94676064 | 5.381880217 |
| SW253 | Bi-parental | IR 50::C1 | 1.314752243 | -4.829462338 |
| SW257 | Bi-parental | IR00A107 | 0.935538238 | 0.647779122 |
| SW258 | Bi-parental | IRRI 105 (PSB Rc18) | 10.84494987 | 9.92223704 |
| SW262 | Bi-parental | IRRI 143 | 3.440094745 | -23.01357698 |
| SW265 | Bi-parental | IR 04A427 | 12.13684995 | 4.57856436 |
| SW268 | Bi-parental | IR 65620-192-3-3-3-2 | 4.997562506 | -2.189285546 |
| SW269 | Bi-parental | IR 68058-71-2-1 | 10.84547309 | 9.738023296 |
| SW270 | Bi-parental | IR 68544-29-2-1-3-1-2 | 14.4092718 | 5.587133544 |
| SW275 | Bi-parental | IRRI 148 (Sahod Ulan 1) | 6.627781797 | -4.695731697 |
| SW277 | Bi-parental | IR28 | 7.129189025 | -12.75931358 |
| SW279 | Bi-parental | IR05N229 | 0.62327474 | -7.648549977 |
| SW298 | Bi-parental | Matatag 3 | 0.654991939 | -9.343967372 |
| SW322 | Bi-parental | IR08N210 | 5.01947077 | -3.589694455 |
| SW368 | Bi-parental | PSB Rc18-Sub1 | 9.472602023 | 5.926370867 |
| SW372 | Bi-parental | IR64 | 1.35176608 | -25.66979085 |
| SW39 | Bi-parental | IR02A127 | 3.108983857 | -2.710307786 |
| SW41 | Bi-parental | IR04A115 | 2.119667884 | -2.210226143 |
| SW43 | Bi-parental | IR05N412 | 9.415261859 | -5.362770266 |
| SW44 | Bi-parental | IR06A150 | 3.063263767 | -3.157807835 |
| SW45 | Bi-parental | IR06N209 | 9.559212219 | -4.747314259 |
| SW46 | Bi-parental | IR07A234 | 6.19267307 | -10.45557893 |
| SW48 | Bi-parental | IR08A172 | 7.480797776 | -3.451501712 |
| SW49 | Bi-parental | IR08A176 | 3.193102841 | -15.75281627 |
| SW50 | Bi-parental | IR08N194 | 9.539138338 | -5.023997344 |
| SW51 | Bi-parental | IR09A136 | 9.321294522 | -9.984108975 |
| SW52 | Bi-parental | IR09A138 | 0.708268261 | -9.821268416 |
| SW53 | Bi-parental | IR09A220 | 6.915307457 | -8.637246054 |
| SW55 | Bi-parental | IR09N190 | 16.37885038 | 9.496371422 |
| SW56 | Bi-parental | IR09N496 | 5.592731008 | -5.186235148 |
| SW57 | Bi-parental | IR09N514 | 1.488936276 | -8.221962948 |
| SW59 | Bi-parental | IR09N531 | 2.009463615 | -10.13594062 |
| SW60 | Bi-parental | IR09N538 | 4.735814566 | -0.562788105 |
| SW61 | Bi-parental | Vandana | 6.393176984 | -8.526430346 |
| SW63 | Bi-parental | IR10N108 | 8.473171374 | 1.292716481 |
| SW69 | Bi-parental | IRRI168 (NSIC Rc 302) | 4.344282462 | -6.863199154 |
| SW70 | Bi-parental | IRRI181 (NSIC 2014 Rc 360) | 3.081119561 | -2.077129851 |
| SW98 | Bi-parental | IRBLta2-RE | 6.162545433 | 2.896190883 |

**Figure S10. 1k-RiCA distribution of F_1_ percentage of similarity between true F_1_ and predicted F_1_**. Percentage of concordance calls between ‘predicted’ F_1_ and F_1_ genotypes generated with the 1k-RiCA in 51 different F_1_ plants. The average concordance estimated value (0.987, or 98.7%) is determined by a dotted line.


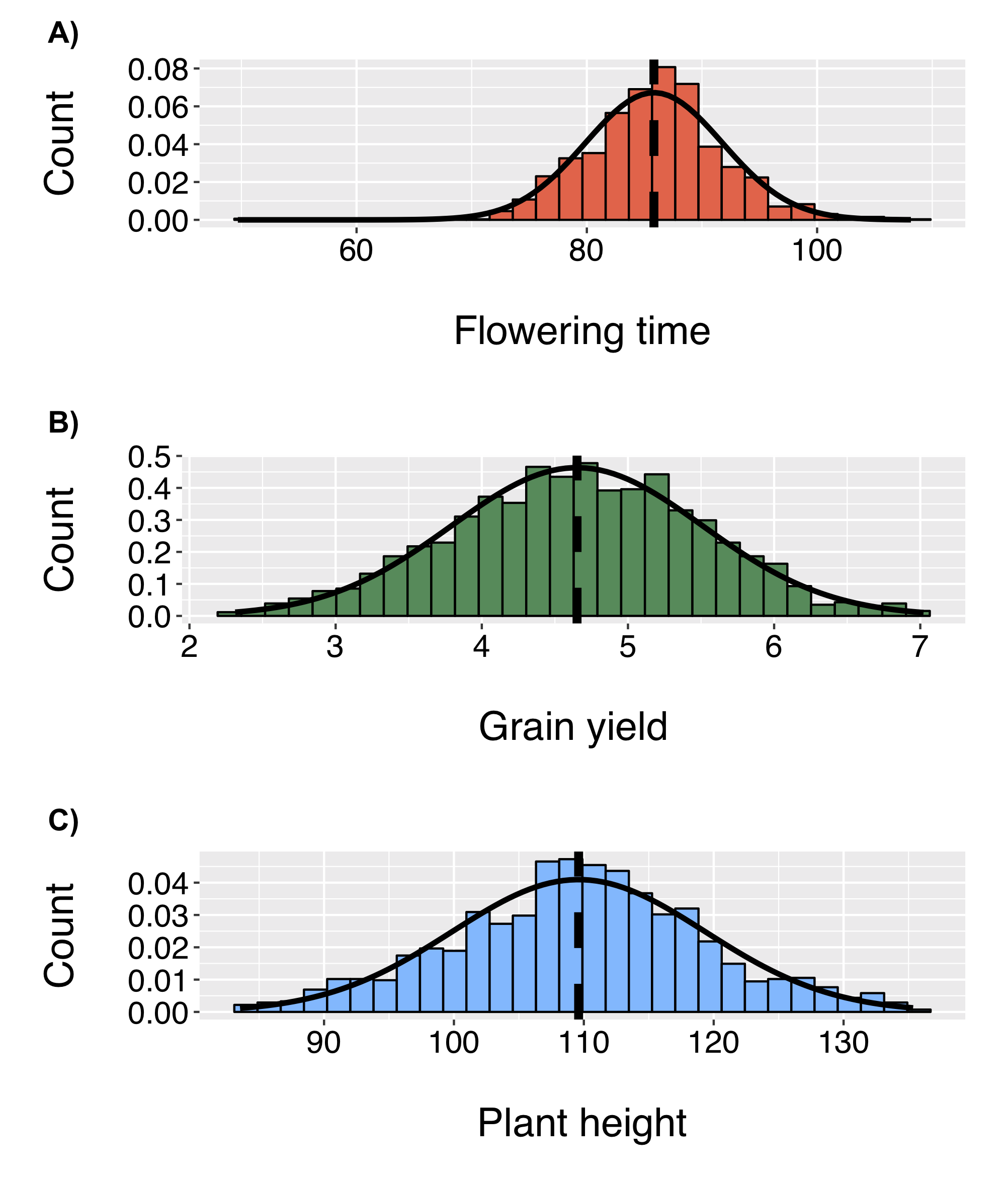


**Figure S11. Phenotypic distribution for flowering time (FLW), grain yield (GY), and plant height (PH)**. Phenotypic distribution for A) FLW, B) GY, and C) PH evaluated in the genomic selection cross validation analysis estimated in 353 elite breeding lines from IRRI’s Favorable Environments Breeding Program.
